# Ada response – a strategy for repair of alkylated DNA in bacteria

**DOI:** 10.1111/1574-6968.12462

**Published:** 2014-06

**Authors:** Damian Mielecki, Elżbieta Grzesiuk

**Affiliations:** Institute of Biochemistry and Biophysics, Polish Academy of SciencesWarszawa, Poland

**Keywords:** Ada response, alkylating agents, Ada proteins, ALKBH, DNA repair

## Abstract

Alkylating agents are widespread in the environment and also occur endogenously. They can be cytotoxic or mutagenic to the cells introducing alkylated bases to DNA or RNA. All organisms have evolved multiple DNA repair mechanisms to counteract the effects of DNA alkylation: the most cytotoxic lesion, *N*^3^-methyladenine (3meA), is excised by AlkA glycosylase initiating base excision repair (BER); toxic *N*^1^-methyladenine (1meA) and *N*^3^-methylcytosine (3meC), induced in DNA and RNA, are removed by AlkB dioxygenase; and mutagenic and cytotoxic *O*^6^-methylguanine (*O*^6^meG) is repaired by Ada methyltransferase. In *Escherichia coli*, Ada response involves the expression of four genes, *ada*, *alkA*, *alkB*, and *aidB*, encoding respective proteins Ada, AlkA, AlkB, and AidB. The Ada response is conserved among many bacterial species; however, it can be organized differently, with diverse substrate specificity of the particular proteins. Here, an overview of the organization of the Ada regulon and function of individual proteins is presented. We put special effort into the characterization of AlkB dioxygenases, their substrate specificity, and function in the repair of alkylation lesions in DNA/RNA.

## Introduction

Alkylating agents are widespread in the environment and are also produced endogenously, as by-products of cellular metabolism. They introduce lesions into DNA or RNA bases that can be cytotoxic, mutagenic, or neutral to the cell. Cytotoxic lesions block replication, interrupt transcription, or signal the activation of apoptosis, whereas mutagenic ones are miscoding and cause mutations in newly synthesized DNA. In mammals, these mutations are thought to be a major mechanism of carcinogenesis, neurodegenerative diseases, and aging.

The major products of alkylation include *N*^7^-methylguanine (7meG), *N*^3^-methyladenine (3meA), and *O*^6^-methylguanine (*O*^6^meG), with smaller amounts of *N*^1^-methyladenine (1meA), *N*^3^-methylcytosine (3meC), *O*^4^-methylthymine (*O*^4^meT), and methyl phosphotriesters (MPT). In terms of cytotoxic and mutagenic effects, 3meA and *O*^6^meG have been found as the most powerful among the 11 identified base modifications.

Endogenously produced *S*-adenosylmethionine (SAM) appears to be one of the main donors of the methyl group, generating 7meG, 3meA, and *O*^6^meG lesions in DNA. However, its reactivity is about 2000-fold weaker than that of methyl methanesulfonate (MMS) (Rydberg & Lindahl, 1982). Because methylation plays a critical role in several cellular processes, any alterations in SAM concentration may affect cell function. Moreover, in higher eukaryotes, abnormalities in SAM metabolism are connected with liver diseases, neurological disorders, and spontaneous carcinogenesis.

Organisms have evolved multiple DNA repair mechanisms to counteract the effects of DNA alkylation. The most cytotoxic lesion, 3meA, is excised by a specific DNA glycosylase, AlkA protein, initiating base excision repair (BER). The mutagenic lesions, 1meA and 3meC, induced in DNA and RNA, are removed by AlkB dioxygenase. Mutagenic and cytotoxic O^6^meG, on the other hand, is repaired by Ada methyltransferase, which transfers the methyl group from the lesion to its own cysteine residue. All three proteins are expressed as part of the so-called adaptive response (Ada response).

## Ada regulon organization

**Fig. 1:**
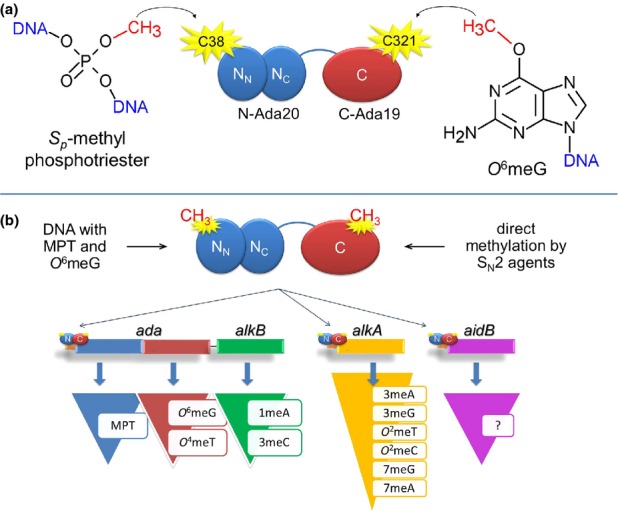
Alkylation damaged DNA is repaired in *E. coli* by four Ada response proteins Ada, AlkA, AlkB, and AidB, while the methylated Ada protein acts as a transcriptional activator in their synthesis. (a) Domain organization of Ada protein and reactions performed by the N-Ada20 and C-Ada19 domains. (b) Ada regulon activation and the activity of particular proteins. Ada protein is methylated at its C38 and C321 residues by transferring the methyl moiety from damaged DNA or by direct action of alkylating agents. Methylated Ada (indicated by yellow stars) acts as a transcriptional activator of three operons: *ada-alkB*, *alkA*, and *aidB*. Synthetized proteins: Ada, AlkB, AlkA, and AidB act on indicated DNA lesions; the substrate(s) for AidB protein has not been confirmed yet.

In *Escherichia coli*, the induction of the Ada response with alkylating agents results in increased expression of four genes: *ada*, *alkA*, *alkB,* and *aidB* (Lindahl *et al*., 1988), regulated by *ada*-encoded Ada protein. *Ada* transcription is activated by methylated Ada protein, whereas unmethylated protein, present in *E. coli* cells in the amount of >200 molecules negatively regulates transcription from the *ada* promoter (Fig. 1).

Although profoundly characterized, the organization of *E. coli* Ada regulon differs from that described in *Pseudomonas putida*. In *E. coli,* the *ada* and *alkB* genes comprise one operon, separated by 160 kbp from *alkA*, whereas in *P. putida*, the *alkA* and *ada* genes are located side-by-side and are transcribed in the *alkA-ada* direction, while the *alkB* gene is located about 3 Mbp away. Additionally, the predicted amino acid sequences of *P. putida* KT2440 and *E. coli* K12 substr. DH10B Ada proteins exhibit 54.6% identity (Mielecki *et al*., 2013).

## Ada response proteins

### Ada methyltransferase

**Fig. 2:**
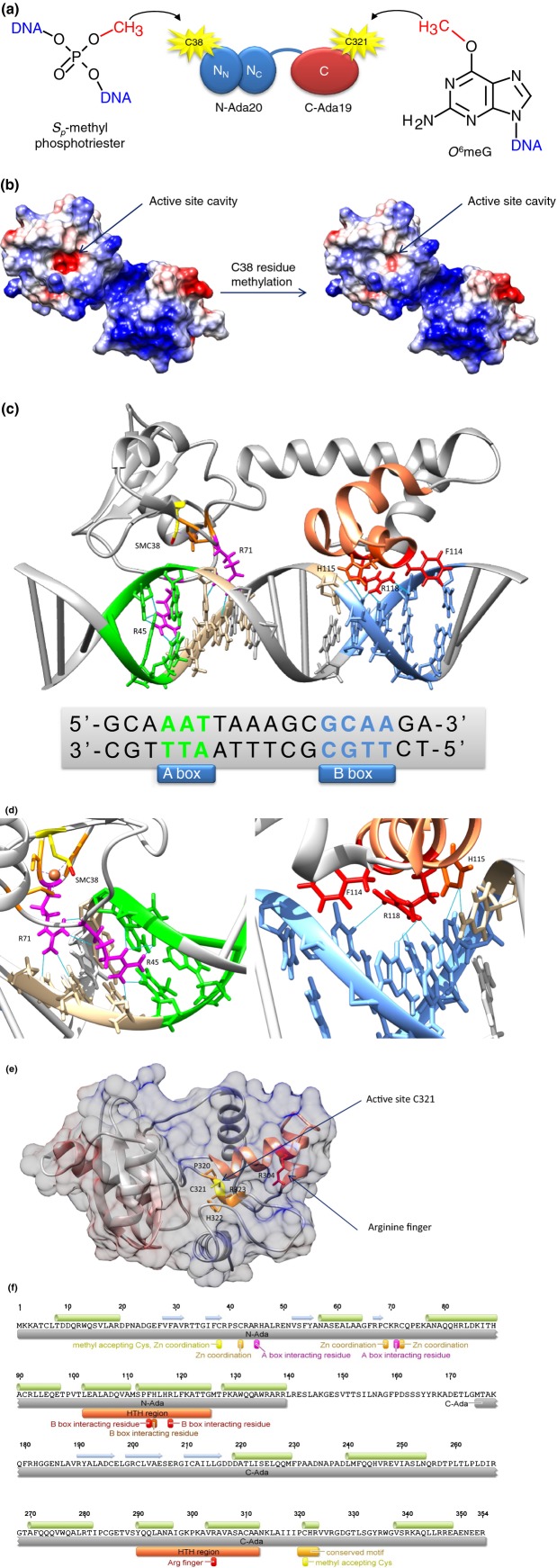
The activity of Ada methyltransferase. (a) The Ada protein acts as a methyltransferase accepting aberrant methyl residues from methyl phosphotriesters (MPT) and *O*^6^-methylguanine (*O*^6^meG) to its C38 and C321 residues, respectively, in suicidal reaction. (b) The active site of N-Ada20 domain (PDB: 1ZGW: He *et al*., 2005) turns almost neutral after C38 residue methylation abolishing negative charge clashes with DNA backbone. (c) The methylated N-Ada20 (PDB: 1ZGW) starts to act as a transcriptional activator interacting specifically with A and B boxes in the promoter regions of *ada*, *alkA*, and *aidB* genes. (d) The N-terminal R45 and R71 form extensive hydrogen bonding with A/T residues of A box, and the C-terminal F114, H115, and R118 of N-Ada20 domain contact B box residues through hydrogen bonds as well as hydrophobic interactions. (e) The C321 catalytic residue of C-Ada19 (PDB: 1SFE: Moore *et al*., 1994) is hidden in the domain structure and together with P320, H322, and R323 forms a highly conserved motif. The conformational changes are indispensable for the occurrence of methyltransferase reaction. The R304 is responsible for nucleotide base flipping. (f) Also the specific residues and domains of Ada protein (below sequence) as well as its secondary structure elements (above sequence) are shown. Fig. 1b, c, d, and e were prepared with UCSF Chimera package (Pettersen *et al*., 2004). Fig. 2f was produced in Geneious 7.1.4 (Biomatters, http://www.geneious.com).

The *E. coli* Ada protein is a chemosensor directly repairing methylated bases and coordinating the resistance response to methylating agents (Ada response). The protein is composed of two major domains: a 20 kDa N-terminal domain (N-Ada20) and a 19 kDa C-terminal domain (C-Ada19), linked by a hinge region susceptible to proteolytic cleavage. The N-Ada20 repairs the *S*_*p*_-diastereoisomers of the MPT lesion in DNA by transferring the methyl group onto its C38 residue (Lindahl *et al*., 1988). The C-Ada19, on the other hand, repairs the highly mutagenic O^6^meG and O^4^meT by transferring the methyl group onto its C321 residue. The mechanism of action of both *E. coli* Ada domains is suicidal because the methyl transfer is irreversible (Fig.2a).

The question arises: how does C38 methylation, a relatively small modification, render the Ada protein a strong transcriptional activator? According to the previously proposed hypothesis, this methylation causes conformational changes promoting DNA–protein interaction. However, recent work by He *et al*. (2005) revealed that the simple ‘electrostatic switch’ is responsible for hundredfold enhancement of Ada affinity to DNA (Fig.2b). In the unmethylated form, there exists an electrostatic repulsion between the DNA phosphate backbone oxygens and the thiolate groups of C38 and C69. The methylation of C38 diminishes the relatively high negative charge of the active site, consequently allowing sequence-specific Ada–DNA interaction.

A full view of Ada interactions with the *ada* promoter has been provided by structural analysis of the protein–DNA complexes. Although they are at different distances from transcription start sites, all three promoters, *ada*, *alkA*, and *aidB*, contain two short sequences: A box (AAT) and B box (GCAA). The A/T composition of the A box is crucial because these base residues form hydrogen bonds with the guanidinium group of Ada R45, and its main chain amide hydrogen bonds the A/T base pair just downstream of the A box. The R71 makes analogous interactions with two subsequent A/T base pairs. On the other hand, the methyl groups of the A/T pairs of the B box interact with the phenyl ring of F114 and the distance between the guanidinium group of R118 and *O*^6^ atoms of G/C pairs is sufficient for hydrogen bonds to be formed. Additionally, these interactions are probably stabilized by the hydrogen bonds between the H115 residue and a C base upstream from the B box (He *et al*., 2005). This phenomenon could explain why Ada exhibits about a 10-fold higher affinity for *ada* than *alkA* and *aidB* promoters, which both have an A nucleotide residue at the corresponding positions (Fig.2c, d and f) (Landini & Volkert, 1995).

To make C321 available to the substrate, a conformational change is needed because the active site thiol of this residue, in the PCHR motif, is hidden in the structure of C-Ada19 (Fig.2e and f) (Moore *et al*., 1994). One model suggests a rotation of the C-terminal helix, resulting in the exposition of DNA binding surface of the protein. Another model assumes that the C-terminus of C-Ada19 forms α-helices and connecting loops (Katayanagi *et al*., 1990). Three helices show strong similarity to the helix-turn-helix (HTH) motif involved in binding to DNA. On the other hand, the second helix of the HTH motif taking part in DNA binding contains a conserved RAV[A/G] sequence, termed ‘arginine-finger,’ promoting base flipping of the substrate DNA nucleotide (Daniels *et al*., 2000).

The main role for C-Ada19 is to provide the interaction site for Ada protein with RNA polymerase (RNAP). Ada, as a transcription factor, requires an interaction with the C-terminal α subunit of RNAP, at least in the case of the *ada* promoter. Considering this feature, Ada has been classified as a type I transcription factor. RNAP binds to the *ada* promoter only in the presence of methylated Ada protein. Its σ factor is essential to start transcription activated by the Ada protein. Based on these findings, a model of Ada activated transcription from *ada* and *aidB* promoters has been proposed (Landini *et al*., 1998). Meanwhile, the mode of RNAP and Ada action at the *alkA* promoter differs from that described previously. At the *alkA* promoter, the RNAP binds to DNA sequence very weakly and both forms of Ada are able to stimulate the expression from the *alkA* promoter. Additionally, the RNAP α subunit binding to *alkA* promoter requires Ada protein.

In *E. coli* in the stationary phase of growth, promoters of the adaptive response genes, *ada*, *alkA*, and *aidB*, are regulated differently. All three are induced slightly better by transcription factor σ^s^ when DNA is not damaged. Surprisingly, in the presence of methylated Ada protein, a strong σ^s^-dependent induction of *aidB* promoter has been observed, while the transcription from *alkA* promoter was stronger in the absence of the σ^s^ factor (Landini & Busby, 1999).

*E. coli* cells in an un-induced state contain about two to three molecules of Ada protein, whereas after induction of the Ada response this number increases to about 3000 molecules per cell. In the *E. coli ada*^−^ mutant, alkyltransferase activity is still observed, suggesting the presence of another enzyme of similar activity. Indeed, the constitutively expressed 19 kDa Ogt protein has been identified as the second alkyltransferase. It shows similar activity as the C-Ada19 domain of Ada; therefore, it repairs O^6^meG and O^4^meT in DNA.

### AlkA glycosylase

**Fig. 3:**
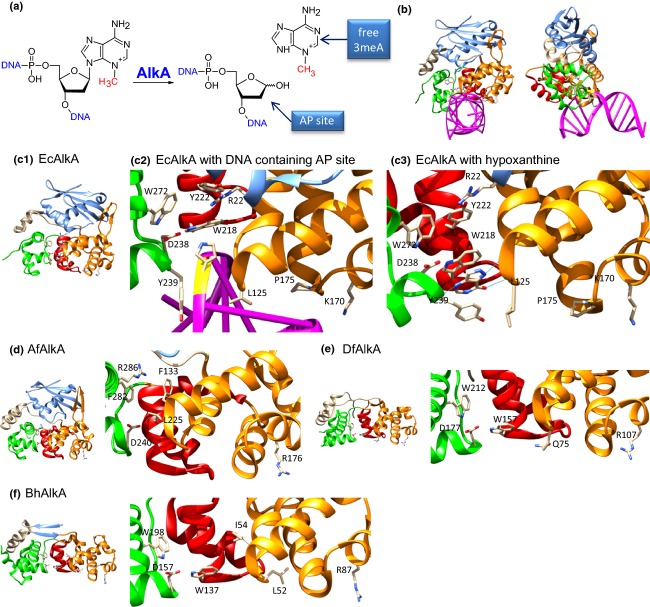
The activity of AlkA glycosylase. (a) The AlkA glycosylase catalyzes the hydrolysis of N-glycosidic bond consequently removing alkylated base from DNA leaving behind the apurinic/apyrimidinic (AP) site further processed within the base excision repair (BER) pathway. (b) During the reaction the 60° DNA bend occurs (PDB: 1DIZ: Hollis *et al*., 2000). (c) The *E. coli* AlkA glycosylase (EcAlkA) is composed of domain I (blue), domain II (orange) containing HhH motif (red), and domain III (green and tan) (c1). The crucial active site residues are shown, catalytic D238 and probable substrate binding W272 and Y222 (c2) (PDB: 1DIZ). There are some ambiguities referring to these hydrophobic residues as one of the EcAlkA structures shows hypoxanthine ligand bound by W218 and Y239 (c3) (PDB: 1PVS: Teale *et al*., 2002). (d and e) It has been confirmed that *Archaeoglobus fulgidus* (AfAlkA) (PDB: 2JHN: Leiros *et al*., 2007) as well as *Deinococcus radiodurans* (DrAlkA) (PDB: 2YG9: Moe *et al*., 2012) glycosylases can repair 1meA and 3meC, canonical AlkB substrates. This phenomenon is not directly linked to the domain I present in AfAlkA, in contrast to DrAlkA. (f) *Bacillus halodurans* (BhAlkA) glycosylase structure (PDB: 2H56) contains partial domain I. Despite differences in the composition of DNA base binding residues in the active site, all three proteins, AfAlkA, DrAlkA, and BhAlkA, include the catalytic aspartate amino acid. Fig. 3b, c1, c2, c3, d, e, and f were prepared with UCSF Chimera package (Pettersen *et al*., 2004).

In *E. coli* cells, there are two enzymes that repair 3meA: the constitutively expressed 3meA DNA glycosylase I (Tag protein) and induced as part of the Ada response 3meA DNA glycosylase II (AlkA protein). Both glycosylases remove 3meA from DNA, leaving behind apurinic/apyrimidinic (AP) sites, subsequently repaired via the BER pathway (Fig.3a). However, the substrate specificity of AlkA is much broader than that of Tag; in addition to 3meA, AlkAs of various prokaryotic and eukaryotic organisms also remove *N*^7^-methyladenine (7meA), 3meG, 7meG, 1,*N*^6^-ethenoadenine (*ε*A), products of nitrosation, for example hypoxanthine (Hx) and oxanine, and some other types of alkylated bases. Further, AlkA, but not Tag, can also remove normal bases (mainly G) from DNA (Berdal *et al*., 1998).

The AlkA protein belongs to the helix-hairpin-helix (HhH) superfamily of DNA glycosylases. The *E. coli* AlkA is built of three domains: the N-terminal domain I with five-stranded β-sheet flanked by two α-helices; domain II, a package of seven α-helices; and domain III consisting of a three-helix bundle with an additional α-helix coming from between domains I and II. The active site lies between the cleft of domains II and III. The activity of AlkA causes 60° DNA backbone bending (Fig.3b and c1) (Labahn *et al*., 1996; Hollis *et al*., 2000). The indispensable catalytic residue is D238 (Fig. 3c2). Although there is no AlkA bound to dsDNA bearing methylated base, structures of AlkA associated with DNA containing AP site or free hypoxanthine have been obtained (Fig.3c2 and c3). Still ambiguity remains which residues are responsible for substrate binding. For example, the AlkA-hypoxanthine structure shows the engagement of W218 and Y239 (PDB: 1PVS; Teale *et al*., 2002) but other structures suggest W272 and, to a lesser degree, Y222, instead (PDB: 1DIZ; Hollis *et al*., 2000). Disregarding this uncertainty, the AlkA active site is generally formed by aromatic amino acid residues, possibly binding damaged base by π–π stacking. The catalytic aspartate, as well as aromatic residues, can also be found in the active sites of AlkA proteins of other *Bacteria*: *Archaeoglobus fulgidus* (AfAlkA), *Deinococcus radiodurans* (DrAlkA), and *Bacillus halodurans* (BhAlkA; Fig.3d, e and f) (Leiros *et al*., 2007; Moe *et al*., 2012). Surprisingly, it has been shown that AfAlkA and DrAlkA proteins can also remove from DNA 1meA and 3meC, which are typically substrates of AlkB dioxygenase; unfortunately, at present, there is no data for BhAlkA. Moe *et al*. (2012) imply that this widened DrAlkA substrate specificity results from the relatively broad binding pocket and, consequently, a ‘highly accessible’ active site. This feature could be the result of the lack of domain I, which imposes structural constraints in EcAlkA. However, this hypothesis seems to be true only for DrAlkA protein, because AfAlkA shows the presence of domain I but is still able to remove 1meA and 3meC bases from DNA.

Our latest study (Mielecki *et al*., 2013) indicates that the amino acid sequence identity between EcAlkA and the AlkA protein of *P. putida* (PpAlkA) is only 17.2%. PpAlkA is shorter by 61 amino acids at its N-terminus; thus, it lacks the N-terminal α/β domain present in EcAlkA. Nevertheless, it still consists of the C-terminal glycosylase domain and the vital catalytic aspartate residue, and exhibits 33.5% sequence identity with DrAlkA. We have found that *P. putida alkA* promoter activity is strongly induced upon mutagen treatment, contrary to the *alkB* promoter, and that AlkA glycosylase protects *P. putida* cells against the cytotoxic action of alkylating agents much more effectively than *E. coli* AlkA.

## AlkB dioxygenases

**Fig. 4:**
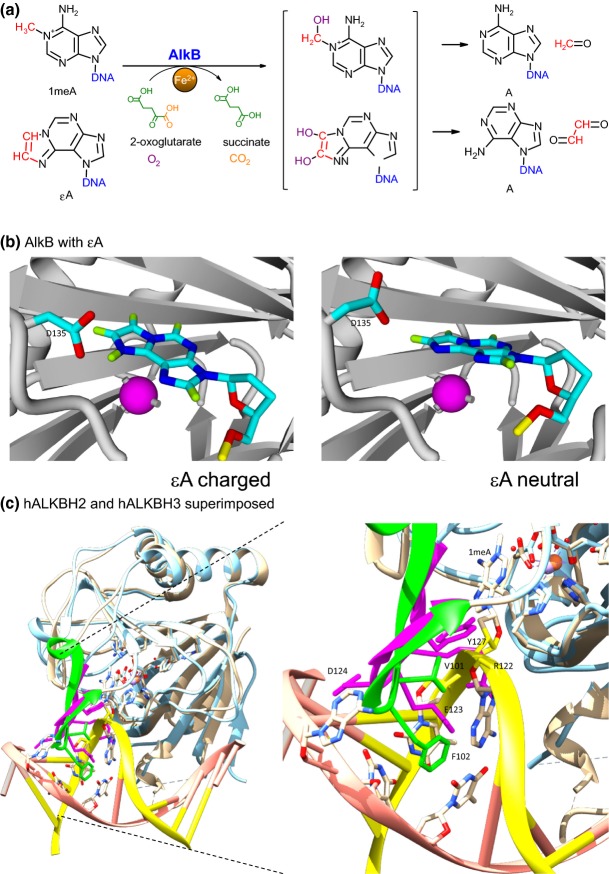
The activity of AlkB dioxygenase. (a) Mechanism of alkylated DNA repair catalyzed by AlkB protein. The aberrant carbon atoms: methyl group in *N*^1^-methyladenine (1meA) and ethylene bridge in 1,*N*^6^-ethenoadenine (εA) are hydroxylated by AlkB. This hydroxylation is exerted with two co-substrates, 2-oxoglutarate (2OG) and molecular oxygen (O_2_), and a co-factor, Fe (II), and leads to the spontaneous release of corresponding aldehydes. (b) The D135 AlkB residue plays a role in the preference for binding protonated substrates, for example εA creates hydrogen bonds with D135 (Maciejewska *et al*., 2013). (c) ALKBH2 (PDB: 3BUC, tan, Yang *et al*., 2008) and ALKBH3 (PDB: 2IUW, light blue, Sundheim *et al*., 2006) exert preference for dsDNA and ssDNA, respectively, although, superimposed, they show almost no difference in 3D structures. This preference is based on the corresponding β-hairpins: the ALKBH2 β-hairpin (green) contains V101 and F102 intercalating into DNA strand stabilizing the distorted double helix and flipping out the base on the complementary strand in the position −1 relative to the aberrant 1meA residue. On the other hand, the ALKBH3 β-hairpin (magenta) bears residues that would promote charge repulsion (E123, D124) as well as structural constraints (R122, Y127) with DNA backbone. Fig. 4c was prepared with UCSF Chimera package (Pettersen *et al*., 2004).

*E. coli alkB-*encoded AlkB (EcAlkB) protein belongs to the dioxygenase superfamily and requires nonheme Fe^2+^ and cofactors, 2-oxoglutarate (2OG) and oxygen (O_2_), to perform oxidative demethylation of DNA/RNA bases (Aravind & Koonin, 2001). AlkB catalyzes the hydroxylation of the methyl group resulting in the formation of succinate and CO_2_ and the restoration of the native bases in the DNA (Fig.4a).

The 1meA and 3meC base modifications are more plausible substrates for AlkB protein when present in ssDNA, because in dsDNA, the ring nitrogens at the *N*^1^ and *N*^3^ positions are protected by hydrogen bond formation. Until the discovery of AlkB function, the mechanism of 1meA and 3meC repair was unknown. Further, AlkB can also revert bulkier adducts such as ethyl, propyl, and hydroxyalkyl groups, as well as exocyclic and ethano adducts. It has also been reported that AlkB removes methyl groups from 1meG and 3meT, but much less efficiently (Delaney & Essigmann, 2004; Maciejewska *et al*., 2010).

**Fig. 5:**
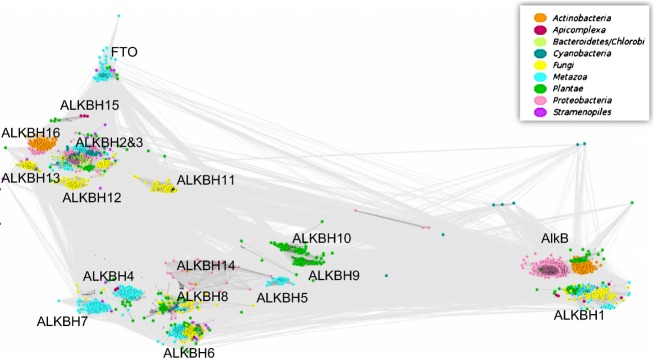
The CLANS clustering analysis of 1943 AlkB protein homologues indicating particular subfamilies (AlkB, ALKBH1-16, FTO), color coded according to taxonomy (Mielecki et al., 2012).

AlkB homologues are present in almost all organisms (Fig.5) (Mielecki *et al*., 2012). Moreover, several dioxygenases can coexist in one cell; thus, questions arise about the specific function of these proteins, especially in eukaryotic cells. Through bioinformatic analysis nine human AlkB homologues were identified, ALKBH1-8 and FTO (Kurowski *et al*., 2003; Gerken *et al*., 2007), all containing a conserved 2OG–Fe(II) dioxygenase domain. Among these homologues, ALKBH1, ALKBH2, ALKBH3, and FTO exhibit methyl moiety oxidation activity, typical for EcAlkB. There is strong evidence that EcAlkB preferentially repairs protonated adducts. The best AlkB substrates, 1meA and 3meC, are most efficiently repaired at physiological pH because in these conditions they exist in a cationic form. Other AlkB substrates are also better repaired at a pH corresponding to their cationic form. Negatively charged D135 is crucial for recognition of the positively charged adduct, located in the proximity of the bound modified base (Fig.4b) (Maciejewska *et al*., 2013).

ALKBH1 exhibits DNA lyase activity at AP sites independent of Fe(II) and 2OG. It also catalyzes methyl group oxidation but at low level (Muller *et al*., 2010; Korvald *et al*., 2012), and modifies the methylation status of histone H2A (Ougland *et al*., 2012). ALKBH2 and ALKBH3, on the other hand, both repair 1meA and 3meC but they differ in selectivity: ALKBH3 as EcAlkB, prefers ssDNA, whereas ALKBH2, dsDNA (Fig.4c) (Sundheim *et al*., 2008; Monsen *et al*., 2010).

Two human homologues: the fat mass and obesity associated FTO protein and ALKBH5, upregulated during hypoxia and involved in spermatogenesis, both catalyze *N*^6^-methyladenine (6meA) demethylation in RNA (Jia *et al*., 2011; Zheng *et al*., 2013). These findings indicate the importance of RNA modification in the regulation of gene expression in health and disease (Jia *et al*., 2013).

Additionally, another human dioxygenase, ALKBH8, exhibits methyltransferase activity modifying uridine in the wobble position of tRNA (van den Born *et al*., 2011).

The AlkB homologues are not expressed equally in different human tissues; for example, a high level of ALKBH2 mRNAs was observed in liver, whereas the expression of ALKBH3 was high in the heart, liver, prostate, and testis (Sedgwick *et al*., 2007). Further, several ALKBHs have been found to be overexpressed in different tumors, suggesting that dioxygenase-directed tumor detoxification may create better conditions for its progression.

Overall, the regulation of ALKBHs expression remains unclear. It is worth mentioning that in eukaryotic cells, the repair of relatively stable mRNA is more important than in prokaryotes (Aas *et al*., 2003). On the other hand, many tRNAs and rRNAs require methylated bases for proper folding and activity. Thus, one can ask, how ALKBHs distinguish between proper and aberrant methylation. Even in *Bacteria*, expression of AlkB proteins is diverse: EcAlkB is induced within the Ada response, whereas in *P. putida* it is expressed constitutively, despite identical activity of both proteins. Moreover, the main AlkB substrates, 1meA and 3meC, are probably both repaired by *P. putida* AlkA glycosylase (Mielecki *et al*., 2013).

## AidB protein

In *E. coli,* AidB is a protein expressed within the Ada response, related in sequence to the acyl-coenzyme A (acyl-CoA) dehydrogenase family (ACADs) (Landini *et al*., 1994). However, its exact role in cell protection against alkylating agents remains unknown. AidB shows weak isovaleryl CoA-dehydrogenase (IVD) activity and exhibits nonspecific binding to dsDNA, suggesting that it may inactivate alkylans before their interaction with DNA.

Rippa *et al*. (2011) have found that the *E. coli aidB*^−^ mutant is as sensitive to MMS as the wild type strain. However, an effect of this mutation has been observed at low, sublethal doses of alkylating agents, indicating an AidB role in DNA protection against by-products of cell metabolism during stationary phase (Volkert *et al*., 1986). This possibility is consistent with the observation of increased level of AidB protein in this phase of growth (Landini *et al*., 1996).

## Concluding remarks

In *Bacteria*, Ada response plays an important role in protecting cells against the cytotoxic and mutagenic action of alkylating agents. In *E. coli*, four genes creating the Ada operon (*ada*, *alkA*, *alkB*, *aidB*) encode four proteins (Ada, AlkA, AlkB, AidB) playing specialized functions in removing alkylating lesions from DNA and RNA. Although they exert their activities exploiting different reaction mechanisms and various structures of catalytic centers, their genes are still controlled within the same regulon. Ada regulon organization and the roles played by the particular proteins differ in individual bacterial species. We discovered that, on the contrary to *E. coli*, *P. putida* AlkB is not induced within Ada regulon but expressed constitutively, and most likely plays a different role than EcAlkB. Further, two Ada response proteins, AlkA and AlkB, differ in importance in protecting *E. coli* and *P. putida* cells against alkylating agents. In the first species, AlkB plays a key role in repairing alkylation lesions, whereas in the latter, AlkA is of the greatest significance. Generally, the multiplicity of AlkB dioxygenases in eukaryotes indicates that they have different functions. The Ada response appears to be of special importance in bacteria that inhabit natural environments because they are exposed to greater cytotoxic/mutagenic action of chemicals, as compared to, for example, intestinal *E. coli*.
